# Successful utilization of nirmatrelvir/ritonavir and dexamethasone in a patient with total artificial heart and COVID-19: A case report

**DOI:** 10.1097/MD.0000000000035464

**Published:** 2023-10-27

**Authors:** Shuroug A. Alowais, Mohammed Bosaeed, Khalid Bin Saleh, Hajar AlQahtani, Nedim Selimovic, Husnat Ahmed, Abdullah A. Alghamdi, Arif Hussain, Hisham A. Badreldin

**Affiliations:** a College of Pharmacy, King Saud Bin Abdulaziz University for Health Sciences, City, Saudi Arabia; b King Abdullah International Medical Research Center, Riyadh, Saudi Arabia; c Pharmaceutical Care Department, National Guard Health Affairs, Riyadh, Saudi Arabia; d King Abdulaziz Medical City, National Guard Health Affairs, Riyadh, Saudi Arabia; e College of Medicine, King Saud Bin Abdulaziz University for Health Sciences, Riyadh, Saudi Arabia.

**Keywords:** nirmatrelvir/ritonavir, paxlovid, total artificial heart, COVID-19, antiviral

## Abstract

**Rationale:**

Management of coronavirus disease 2019 (COVID-19) has been the subject of extensive research and study, leading to the development of strategies and treatments. Nonetheless, there remains a dearth of information concerning patients who require mechanical circulatory system support. This case report presents one of the first documented cases of successful utilization of nirmatrelvir/ritonavir (Paxlovid) and dexamethasone in the treatment of a patient with a total artificial heart.

**Patient concerns:**

The patient in this case study was a 28-year-old male who had been experiencing severe heart failure. In need of a heart transplant, he underwent a procedure for implantation of a total artificial heart as a bridge to transplantation.

**Diagnoses:**

Unfortunately, after the surgical intervention, the patient contracted COVID-19, as confirmed by polymerase chain reaction.

**Interventions:**

The therapeutic approach involved a 5-day regimen of nirmatrelvir/ritonavir at a dosage of 300/100 mg administered twice daily, along with a daily dosage of 6 mg of dexamethasone.

**Outcomes:**

Remarkably, the patient oxygenation level improved on the second day of therapy. Consequently, he was transferred from the intensive care unit to the general floor. After 71 days with the total artificial heart, the patient successfully underwent heart transplantation.

**Lessons:**

This case report provides a compelling example of the successful application of nirmatrelvir/ritonavir and dexamethasone in the treatment of a COVID-19 patient with a total artificial heart. The positive outcome observed in this case underscores the potential use of these therapeutic agents in this specific patient population. However, it is imperative to conduct further research to corroborate and validate these initial findings. This study lays the foundation for further exploration of the efficacy of these drugs in patients with mechanical circulatory support systems.

## 1. Introduction

The first case of COVID-19 was diagnosed in 2019 in Wuhan, China, and the number of cases continued to increase, with the highest risk of mortality observed among patients with comorbid conditions.^[1–3]^ Patients with a history of heart failure had a higher risk of death, mechanical ventilation, and longer hospitalization.^[3,4]^

Management modalities, including drug therapy and devices for patients with advanced heart failure, have evolved significantly over the years, with a focus on improving survival, reducing hospitalizations, and enhancing quality of life. Mechanical circulatory support devices, such as left ventricular assist devices, are used to support heart function in patients with advanced heart failure who do not respond to other treatment modalities.^[5]^

Heart transplantation remains the gold standard treatment for end-stage heart failure in select patients. It is recommended that patients exhaust all other treatment options. Among the different mechanical circulatory support devices that could be used in patients with advanced heart failure is the total artificial heart (TAH) which is a life-saving intervention for patients with advanced heart failure. It involves replacing the failing heart with a sophisticated mechanical device that serves as a bridge to transplantation. This procedure aims to improve the survival rates and enhance the quality of life of patients with end-stage heart failure patients. However, TAH is also accompanied by potential complications and risks, necessitating continued research and optimization to ensure the best possible outcomes for patients undergoing this treatment.^[5]^ Meticulous drug monitoring is crucial for patients after TAH implantation because of alterations in cardiac output, organ perfusion, and drug metabolism; however, data regarding pharmacokinetic modifications of specific drugs in TAH patients are scarce.

In patients with COVID-19, the latest Infectious Diseases Society of America guidelines recommend using 6 mg of dexamethasone intravenously or orally for ten days in hospitalized critically ill patients owing to lower ventilator days and mortality.^[6]^ In addition, paxlovid, a novel antiviral agent, has demonstrated significant potential for managing COVID-19 among high-risk patients. This oral medication combines 2 drugs: nirmatrelvir, a protease inhibitor, and ritonavir, a booster drug used to enhance the effectiveness of other antiviral medications by inhibiting their breakdown via the CYP3A4 enzyme.^[7]^

However, there is currently limited clinical data regarding the administration of nirmatrelvir/ritonavir in patients who have undergone TAH implantation. Further research and case studies are essential to understand the safety and efficacy of this unique patient population better. This case report highlights the therapeutic use of nirmatrelvir/ritonavir in conjunction with dexamethasone for the treatment of a COVID-19-infected patient with a TAH implant. The patient medical history, including relevant comorbidities and prior treatment, was detailed to comprehensively understand the clinical context. The outcomes of this case may contribute to the growing body of knowledge on the management of COVID-19 in patients with TAH implants, and may inform future clinical practice. Therefore, this case report aimed to delineate the clinical trajectory and explore the potential therapeutic ramifications of administering nirmatrelvir/ritonavir in conjunction with dexamethasone in a COVID-19-infected patient who had undergone TAH implantation.

## 2. Case presentation

A 28-year-old male patient with a medical history of myocardial infarction 2 years prior, followed by percutaneous coronary intervention without stenting and insertion of an automatic implantable cardioverter-defibrillator 1 year prior, and known to have ischemic cardiomyopathy with advanced heart failure. The patient was admitted to the hospital on February 25, 2023, because of decompensated heart failure, reduced ejection fraction, and left ventricular thrombus. Biventricular heart failure and intractable ventricular tachycardia were the predominant clinical findings. In addition, he reported experiencing a progressive increase in shortness of breath upon moderate exertion over the past week, accompanied by paroxysmal nocturnal dyspnea. The patient was scheduled to undergo heart transplant workup during admission. He was fully vaccinated against COVID-19, and his admission screening for COVID-19 was negative.

His cardiac condition revealed refractory arrhythmias with extensive LV thrombi and prohibitive pulmonary hypertension. The patient progressively deteriorated and was placed on venoarterial extracorporeal membrane oxygenation (V-A ECMO). The patient was successfully extubated 3 days after ECMO (March 7, 2023). However, the patient developed pyrexia and was started empirically on vancomycin and meropenem. The respiratory cultures were positive for *Klebsiella oxytoca* and *Staphylococcus aureus*. Table 1 shows the culture and susceptibility results. On March 11, the patient was decannulated from VA-ECMO and switched to TAH (SynCardia) as a bridge to transplant candidacy. The patient was successfully extubated the first day after post-TAH implantation.

**Table 1:** Respiratory culture and susceptibility results.

*Klebsiella oxytoca*		*Staphylococcus aureus*	
Trimethoprim/sulfamethoxazole	S	Trimethoprim/sulfamethoxazole	S
Piperacillin/tazobactam	S	Oxacillin	S
Gentamicin	S	Erythromycin	S
Ciprofloxacin	S	Clindamycin	S
Ceftriaxone	S	Cefazolin	S
Ampicillin	R		

R = resistant, S = susceptible.

**Figure 1.**
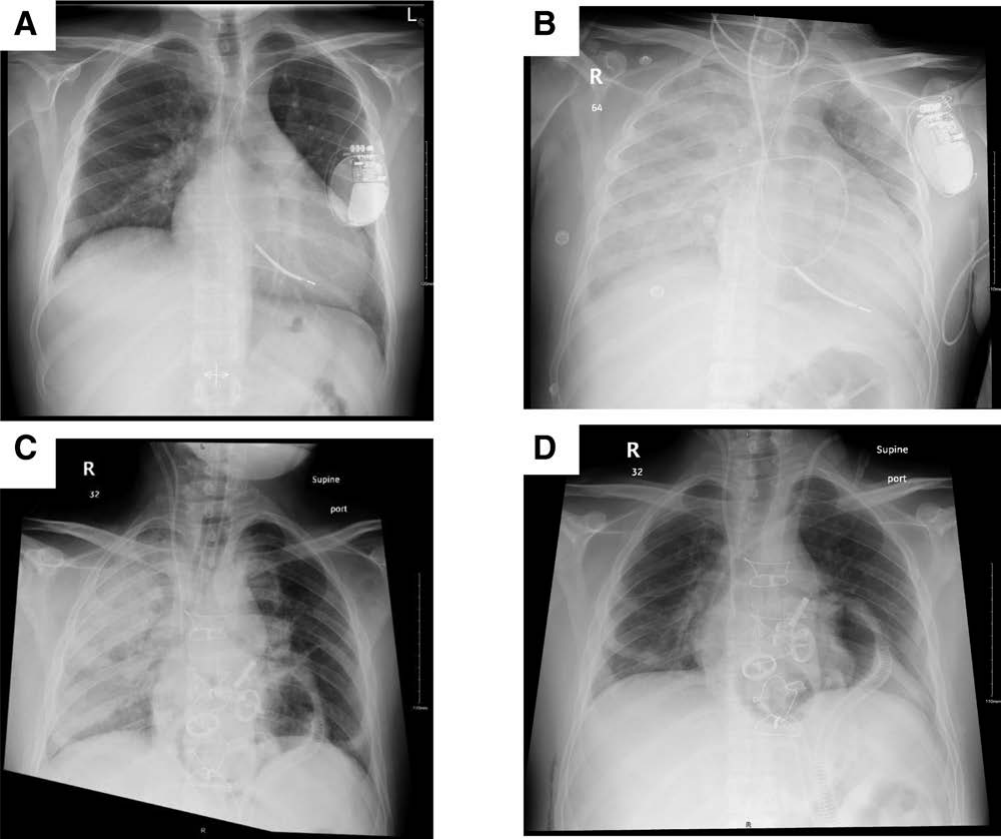
The most relevant chest X-rays during his course of management; (A) Chest X-ray on the day of admission, d 1, February 25, 2023. (B) Chest X-ray just prior to TAH implantation while the patient was on V-A ECMO, d 14, March 10, 2023. (C) Chest X-ray day post TAH implantation, d 15, March 11, 2023. (D) Chest X-ray 1 wk after initiation of Paxlovid therapy; d 24, March 20, 2023.

The patient respiratory status subsequently deteriorated, requiring oxygenation via a high-flow nasal cannula (5 L/min), which prompted the broadening of the antimicrobial regimen in which amikacin and caspofungin were added to the vancomycin and meropenem regimen. The levels of inflammatory markers, including C-reactive protein (337 mg/L) and lactate dehydrogenase (2326 U/L; LDH increased secondary to hemolysis in patients with TAH). Bronchoscopy and bronchoalveolar lavage were performed, and the patient underwent continuous renal replacement therapy. Due to elevated liver function tests, caspofungin was later switched to anidulafungin. The following day, the patient tested positive for COVID-19 in bronchoalveolar lavage samples and was initiated on nirmatrelvir/ritonavir 300/100 mg PO twice daily and dexamethasone 6 mg IV daily, which was changed to dexamethasone 6 mg PO daily after 2 days. Table 2 shows the antimicrobial medications administered per indication and number of hospitalization days. Oxygen requirements started to improve over the subsequent days of antiviral therapy, reaching 3 L/min via a nasal cannula on day 5 of nirmatrelvir/ritonavir. Radiological improvement manifested on repeated chest X-rays taken daily, from interval progressive consolidation on pre-day 1 of nirmatrelvir/ritonavir to improved bibasilar atelectasis by the end of antiviral treatment. Figure 1 shows the most relevant chest radiographs obtained during management.

**Table 2:** Antimicrobial medications per indication and hospitalization days.

Medication	Indication	Hospitalization d
Ceftriaxone	Positive respiratory culture K. oxytoca	d 4–8
Piperacillin/tazobactam	Hospital Acquired Pneumonia (HAP)	d 8–11
Vancomycin	HAP	d 11–23
Meropenem	HAP	d 11–23
Amikacin	HAP	d 16–17
Caspofungin one dose	HAP	d 16
Anidulafungin	HAP	d 17–23
Nirmatrelvir/ritonavir	COVID-19	d 17–21
Dexamethasone	COVID-19	d 17–21

**Figure 2.**
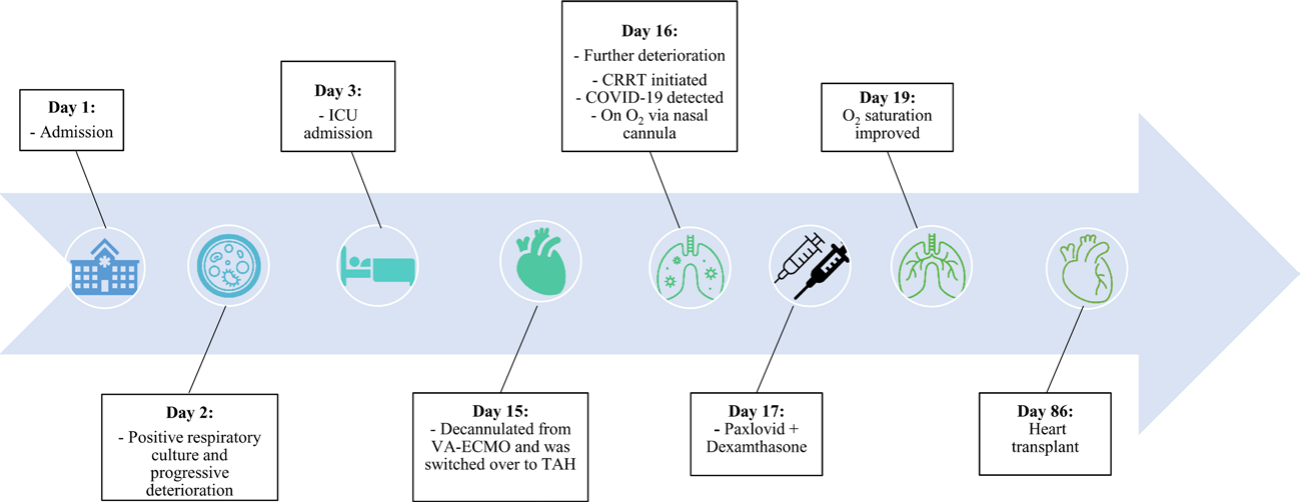
Chronological overview of events and interventions from the point of hospital admission to the heart transplant procedure.

Unfortunately, no chest CT scans have been performed during the COVID-19 pandemic. continuous renal replacement therapy was continued for 5 days, after which the patient renal function improved and he exhibited satisfactory urine output. The patient successfully underwent heart transplantation 10 weeks after TAH support and 5 weeks and 3 days after the onset of COVID-19. The hospitalization timeline is shown in Figure 2.

## 3. Discussion

Treating complicated infections such as COVID-19 in TAH patients poses significant challenges for healthcare professionals given the heightened risk of complications and altered pharmacokinetics due to the presence of TAH, particularly concerning changes in cardiac output. Recently, a case report documented the first instance of COVID-19 in a 27-year-old man with Marfan syndrome and TAH implant. He was diagnosed with COVID-19 2 months post-TAH and developed acute respiratory distress syndrome. However, the patient condition was successfully managed using corticosteroid therapy, prone positioning, and mechanical ventilation.^[8]^ Our patient received nirmatrelvir/ritonavir and dexamethasone and showed significant improvement after 2 days of treatment, with a significant reduction in oxygen requirements and radiological improvement evident on repeated chest X-rays. The patient underwent a successful heart transplantation 38 days after antiviral therapy.

The United States Food and Drug Administration granted emergency authorization for the use of nirmatrelvir/ritonavir in December 2021 and subsequently approved its use in June 2023 for treating mild-to-moderate COVID-19 infections among patients at risk of developing severe complications.^[9]^ The efficacy of nirmatrelvir/ritonavir in managing COVID-19 in high-risk patients is supported by clinical evidence. In a phase 2 to 3 double-blinded, randomized, controlled trial (EPIC-HR), nirmatrelvir/ritonavir remarkably reduced hospitalization in unvaccinated, non-hospitalized patients with COVID-19 or 30-day mortality rates compared to placebo (0.77% vs 7.01%, respectively). In addition, patients receiving nirmatrelvir/ritonavir had a significantly reduced viral load on day 5 compared with the placebo.^[10]^

Furthermore, a large retrospective study comprising 4737 patients corroborated the mortality reduction with nirmatrelvir/ritonavir in most vaccinated patients. Interestingly, immunocompromised patients and patients with cardiovascular disease were among the patients who benefited the most from the drug.^[11]^ However, a randomized open-label trial in China failed to find a 28-day mortality benefit and viral clearance with nirmatrelvir/ritonavir compared to the standard of care in COVID-19 patients with severe comorbidities.^[12]^ Although nirmatrelvir/ritonavir is a relatively new drug with limited safety data, the EPIC-HR study reported adverse effects similar to those observed in the placebo group. However, it is essential to note that insufficient data support the use of nirmatrelvir/ritonavir in critically ill patients, including those who have undergone TAH implantation.

In conclusion, this case report highlights the potential therapeutic benefits of nirmatrelvir/ritonavir-based therapy in a young patient with COVID-19, shortly after TAH implantation. The promising outcome of this case is valuable to the medical literature, given the heightened risk of complications and severe illness associated with COVID-19 in high-risk patients, such as those with TAH implants. Nevertheless, further research is needed to optimize the treatment of such complex cases and to establish the safety and efficacy of this approach in this population.

## Author contributions

**Conceptualization:** Shuroug A. Alowais, Mohammed Bosaeed, Nedim Selimovic, Hisham Badreldin.

**Project administration:** Shuroug A. Alowais, Mohammed Bosaeed, Hajar AlQahtani, Nedim Selimovic, Abdullah A. Alghamdi, Hisham Badreldin.

**Writing – original draft:** Shuroug A. Alowais, Mohammed Bosaeed, Khalid Bin Saleh, Hisham Badreldin.

**Writing – review & editing:** Shuroug A. Alowais, Mohammed Bosaeed, Khalid Bin Saleh, Hajar AlQahtani, Nedim Selimovic, Husnat Ahmed, Abdullah A. Alghamdi, Arif Hussain, Hisham Badreldin.

## References

[1] Wu F Zhao S Yu B. A new coronavirus associated with human respiratory disease in China. Nature. 2020;579:265–9. 32015508 10.1038/s41586-020-2008-3 PMC7094943

[2] Djaharuddin I Munawwarah S Nurulita A. Comorbidities and mortality in COVID-19 patients. Gac Sanit. 2021;35:S530–2. 34929892 10.1016/j.gaceta.2021.10.085 PMC8677356

[3] Saleh KB Hafiz A Alsulaiman K. Clinical characteristics and outcomes of patients with heart failure admitted to the intensive care unit with coronavirus disease 2019 (COVID-19): a multicenter cohort study. Am Heart J Plus. 2021;7:100033. 34308397 10.1016/j.ahjo.2021.100033 PMC8288252

[4] Alvarez-Garcia J Lee S Gupta A. Prognostic impact of prior heart failure in patients hospitalized with COVID-19. J Am Coll Cardiol. 2020;76:2334–48. 33129663 10.1016/j.jacc.2020.09.549 PMC7598769

[5] Heidenreich PA Bozkurt B Aguilar D. 2022 AHA/ACC/HFSA guideline for the management of heart failure: executive summary: a report of the American College of Cardiology/American Heart Association Joint Committee on Clinical Practice Guidelines. Circulation. 2022;145:e876–94. 35363500 10.1161/CIR.0000000000001062

[6] Bhimraj A Morgan RL Shumaker AH. Infectious diseases society of America guidelines on the treatment and management of patients with COVID-19. Clin Infect Dis. 2020:ciaa478. doi: 10.1093/cid/ciaa478. 32338708 10.1093/cid/ciaa478 PMC7197612

[7] U.S. Food and Drug Administration. Available at: https://www.fda.gov/media/155050/download [access date June 7, 2023].

[8] Lutun J Fauvel C Gay A. COVID-19 in a patient implanted with a total artificial heart: a case report. Eur Heart J Case Rep. 2022;6:ytac317. 36245854 10.1093/ehjcr/ytac317 PMC9555052

[9] FDA Approves First Oral Antiviral for Treatment of COVID-19 in Adults. U.S. Food and Drug Administration. Available at: https://www.fda.gov/news-events/press-announcements/ fda-approves-first-oral-antiviral-treatment-covid-19-adults [access date 7, 2023].

[10] Hammond J Leister-Tebbe H Gardner A. Oral nirmatrelvir for high-risk, nonhospitalized adults with Covid-19. N Engl J Med. 2022;386:1397–408. 35172054 10.1056/NEJMoa2118542 PMC8908851

[11] Najjar-Debbiny R Gronich N Weber G. Effectiveness of paxlovid in reducing severe coronavirus disease 2019 and mortality in high-risk patients. Clin Infect Dis. 2023;76:e342–9. 35653428 10.1093/cid/ciac443 PMC9214014

[12] Liu J Pan X Zhang S. Efficacy and safety of PAXLOVID in severe adult patients with SARS-COV-2 infection: a multicenter randomized controlled study. Lancet Reg Health West Pac. 2023;33:100694. 36777445 10.1016/j.lanwpc.2023.100694 PMC9899586

